# Costs of TB services in India

**DOI:** 10.5588/ijtld.21.0105

**Published:** 2021-12-01

**Authors:** S. Chatterjee, M. N. Toshniwal, P. Bhide, K. S. Sachdeva, R. Rao, Y. V. Laurence, N. Kitson, L. Cunnama, A. Vassall, S. Sweeney, I. Garcia Baena

**Affiliations:** 1 George Institute for Global Health, New Delhi, India; 2 University of New South Wales, Sydney, NSW, Australia; 3 Manipal Academy of Higher Education, Manipal, India; 4 Independent consultant, Public Health Division, Durga Clinic, Akola, India; 5 Central TB Division, Ministry of Health and Family Welfare, Government of India, New Delhi, India; 6 Department of Global Health and Development, Centre for Health Economics in London, London School of Hygiene & Tropical Medicine, London, UK; 7 Health Economics Unit & Health Economics Division, University of Cape Town, Cape Town, South Africa; 8 Global TB Programme, World Health Organization, Geneva, Switzerland

**Keywords:** unit cost, pulmonary TB, extrapulmonary TB, first-line treatment, BCG vaccination

## Abstract

**BACKGROUND:**

There is a dearth of economic analysis required to support increased investment in TB in India. This study estimates the costs of TB services from a health systems’ perspective to facilitate the efficient allocation of resources by India’s National Tuberculosis Elimination Programme.

**METHODS:**

Data were collected from a multi-stage, stratified random sample of 20 facilities delivering TB services in two purposively selected states in India as per Global Health Cost Consortium standards and using Value TB Data Collection Tool. Unit costs were estimated using the top-down (TD) and bottom-up (BU) methodology and are reported in 2018 US dollars.

**RESULTS:**

Cost of delivering 50 types of TB services and four interventions varied according to costing method. Key services included sputum smear microscopy, Xpert^®^ MTB/RIF and X-ray with an average BU costs of respectively US$2.45, US$17.36 and US$2.85. Average BU cost for bacille Calmette-Guérin vaccination, passive case-finding, TB prevention in children under 5 years using isoniazid and first-line drug treatment in new pulmonary and extrapulmonary TB cases was respectively US$0.76, US$1.62, US$2.41, US$103 and US$98.

**CONCLUSION:**

The unit cost of TB services and outputs are now available to support investment decisions, as diagnosis algorithms are reviewed and prevention or treatment for TB are expanded or updated in India.

## Abstract (fr)

**CONTEXTE:**

Les analyses économiques requises pour favoriser l’accroissement des investissements destinés à la lutte contre la TB en Inde font cruellement défaut. Cette étude a estimé les coûts des services antituberculeux du point de vue des systèmes de santé afin de faciliter l’allocation efficace des ressources par le programme national d’élimination de la tuberculose indien.

**MÉTHODES:**

Les données ont été recueillies auprès d’un échantillon aléatoire, stratifié à plusieurs niveaux de 20 établissements proposant des services antituberculeux dans deux états indiens intentionnellement sélectionnés conformément aux normes du Global Health Cost Consortium, à l’aide du Value TB Data Collection Tool. Les coûts unitaires ont été estimés à l’aide de la méthode descendante (TD) et ascendante (BU), et sont rapportés en dollars américains 2018.

**RÉSULTATS:**

Le coût d’une offre de 50 types de services antituberculeux et de quatre interventions variait en fonction de la méthode d’évaluation des coûts utilisée. Les services clés comprenaient l’examen microscopique des frottis d’expectorations, le test Xpert^®^ MTB/RIF et la radiographie pulmonaire, avec des coûts BU moyens respectifs de 2,45 $ US, 17,36 $ US et 2,85 $ US. Le coût BU moyen pour la vaccination par le bacille de Calmette-Guérin, la recherche passive de cas, la prévention de la TB chez l’enfant de moins de 5 ans par isoniazide et le traitement de première ligne des nouveaux cas de TB pulmonaire et extra-pulmonaire était respectivement de 0,76 $ US, 1,62 $ US, 2,41 $ US, 103 $ US et 98 $ US.

**CONCLUSION:**

Le coût unitaire des services et produits antituberculeux est désormais disponible pour étayer les décisions d’investissement, au moment où les algorithmes diagnostiques sont revus et où la prévention ou le traitement de la TB s’étendent ou sont actualisés en Inde.

India has high burden of TB and multidrug-resistant TB (MDR-TB).^1^ There were 2.64 million incident TB (including HIV-positive) cases in 2019, a rate of around 193 per 100,000 (uncertainty interval 132–266) population.^1^ Drug resistance levels are estimated at respectively 2.8% (uncertainty interval 2.3–3.5) and 14% (uncertainty interval 14–14) among new and retreatment cases. India has developed an ambitious plan for ending TB by 2025, 5 years before the global target.^2^ To do so, the national TB control programme has undergone a paradigm shift in recent years, including near complete nationwide web- and case-based notification of all TB cases (including cases in private sector care), initiation of first-line treatment (FLT) for 94% of notified drug-susceptible TB (DS-TB) patients, expansion of the laboratory network, adoption of WHO-endorsed rapid molecular diagnostics^3^ and improved private sector engagement. India’s national TB budget in 2020 is almost double what it was in 2016, and domestic funding for this budget in 2020 is 3.7 times the level it was in 2016.^1^

Investment cases, financial planning or resource allocation and economic analysis all require an understanding of the costs of TB services in India. Two recent literature reviews identified five studies with primary cost data for TB in India from a provider perspective.^4^,^5^ Two studies estimated provider cost of public and private partnership in DOTS delivery, two estimated diagnostic costs and the other estimated costs in 2002,^6^–^10^ indicating a clear dearth of provider cost data of TB services in India.

Our study aimed to estimate costs of TB services from a health systems’ perspective following latest costing standards^11^ to enable analyses that support India’s National Tuberculosis Elimination Programme (NTEP) to allocate their resources in an efficient way.

## METHODS

Methods for data collection for estimating unit costs of 50 TB services and four interventions (bacille Calmette-Guérin [BCG] vaccination, passive case-finding [PCF] in adults with pulmonary TB (PTB) and extrapulmonary TB [EPTB], TB prevention in children under 5 years with isoniazid and first-line drug treatment [FLT]) were adapted from “Costing Guidelines for Tuberculosis Interventions”.^11^ Other TB-related interventions such as active case-finding, intensive case-finding, screening, cough triage and second-line drug treatment were either not delivered or were partially delivered in the sampled facilities during the study period. The costs of the partial interventions are not presented in this paper; however, costs of individual direct and ancillary services comprising these interventions will be made available as part of the full dataset to enable future research. Costs were estimated from a health provider’s perspective. Full financial and economic costs were collected retrospectively and reflected ‘real world’ implementation of interventions. Time horizon was one patient episode of care. Outputs and interventions which were not fully implemented in health facilities at the time of data collection were removed from the analysis. No start-up costs for new interventions or costs of supporting change (e.g., pilots or technical assistance) were included. Estimation of future savings, above service level costs, research costs and other unrelated costs were excluded.

### Sampling

Data were collected from a multi-stage, stratified random sample of 20 facilities delivering routine TB services in two purposively selected districts in two states in India: Pune in Maharashtra and Madurai in Tamil Nadu. In the light of study budget limitations, the NTEP purposively selected two districts in two states based on population distribution across urban, rural, tribal/hard to reach and slum settings; presence of drug-resistant TB centre, intermediate reference laboratory (IRL)/culture and drug susceptibility testing (DST) laboratory, and Xpert^®^ MTB/RIF (Cepheid, Sunnyvale, CA, USA) in the government sector; presence of medical college (private and government sectors); as well as private sector facilities such as designated microscopy centres (DMCs), DOT centres, antiretroviral therapy centre; and in general, the presence of a wide range of TB interventions or services.

The sampling frame was created from the electronic national list of healthcare facilities and laboratories registered in *Nikshay* (nationwide web- and case-based notification of cases to the NTEP).^12^ Inclusion criterion was health facilities that provided TB treatment or diagnosis. Exclusion criterion was prisons where TB services were provided. A multistage, stratified random sampling approach was adopted for selecting anonymised facilities within each of the two districts. The final sample considered in this study is given in Table 1.

**Table 1:** Characteristics of sampled sites delivering TB activities in Pune, Maharashtra; and Madurai, Tamil Nadu, India

Facility type	Rural facilities *n*	Urban facilities *n*	Public facilities *n*	Private for profit facilities *n*	Total number of sites	Total number of patients initiated on TB treatment in 2018
Health centre	3	2	5	0	5	210
Primary hospital	2	2	3	1	4	312
Secondary hospital	1	6	3	4	7	447
Tertiary hospital	0	2	1	1	2	669
Laboratory	0	2	1	1	2	NA
Total	6	14	13	7	20	1,638

NA = not applicable.

### Data collection

Cost data were retrospectively collected by two trained researchers over a period of a year in 2018–2019 using publicly available Value TB standard tools and checklists in line with Global Health Cost Consortium’s reference case.^13^ All costs were collected for the financial year 2017–2018 in Indian rupees (INR) and converted into 2018 US dollars using the midmarket average exchange rate from 1 April 2017 to 31 March 2018 (US$1= INR64.456). Data were collected for the financial year 2018–2019 for one facility, as 2017–2018 data were not available; costs in INR were thus deflated using India’s gross domestic product deflator from 2018 to 2017 and were then converted to US$.^14^

Main cost categories were personnel, drugs and supplies, travel and transport, training, other recurrent expenses, and annual value of capital expenditures such as building and equipment. Data were collected from financial records, service statistics reports, and using time sheets, interviews and observation of activities (the latter selected in preference). The price of equipment and consumables were collected from the procurement price list of the study facilities (Supplementary Table S1). If prices of equipment were not available from the study facility, the market price was taken into account. Wastage rates for medical supplies and consumables were assumed at 10%, while for drugs this was 8% (Supplementary Table S1). Staff time spent on different activities were gathered from time sheets, interviews and observation of activities (Supplementary Table S2).

### Analysis

Data were entered into Value TB Data Entry Tool designed in Microsoft Excel (Microsoft, Redmond, WA, USA) as part of the costing tool suite of Costing Guidelines for Tuberculosis Interventions.^11^ Data were pooled, cleaned and analysed using standard programmes in Stata (part of the Costing Tool Suite) (Stata, College Station, TX, USA). Results generation used Stata v15 with exports and visuals in Microsoft Excel.

### Ethics

The study received ethical clearance from the Institutional Ethics Board of the George Institute for Global Health, New Delhi, India, as well as from the London School of Hygiene & Tropical Medicine Research Ethics Committee. The study was also approved by the Health Ministry’s Screening Committee, Ministry of Health and Family Welfare, Government of India; New Delhi, India.

## RESULTS

### Facility characteristics and activity volume

Out of 20 sampled facilities, one public secondary hospital and one public laboratory provided TB services exclusively; all other sampled facilities provided both TB and non-TB services. Detailed characteristics and activity of study facilities are provided in Supplementary Table S3.

### Cost of 50 TB services

Unit costs of the 10 most commonly performed TB services in sampled facilities are given in Table 2 and cost drivers of bottom-up (BU) unit costs are shown in the Figure. For an outpatient screening visit, staff cost was the main cost driver, while for an Xpert test, HIV rapid test and X-ray (using film), it was the consumables. Overhead costs also accounted for a high proportion of services for the remaining six most commonly performed TB services (Figure).

**Figure:**
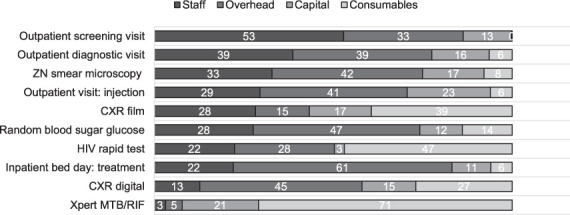
Cost drivers of bottom-up unit costs (%). ZN = Ziehl-Neelsen; CXR = chest X-ray.

**Table 2:** Unit costs of most commonly performed TB services (2018 US$) ^*^

TB services	Facilities *n*	Top-down		Bottom-up	
	
		Mean	95% CI	Mean	95% CI
Smear microscopy ZN	14	4.08	2.42–5.73	2.45	1.50–3.41
CXR digital	9	5.14	1.88–8.40	2.85	1.24–4.46
CXR film	4	3.29	2.73–3.85	1.95	1.59–2.32
HIV rapid test	12	1.97	1.51–2.44	1.30	1.01–1.60
Random blood sugar	15	1.33	0.91–1.75	0.82	0.56–1.07
Xpert MTB/RIF	2	29.81	5.59–54.03	17.36	14.74–19.98
Outpatient diagnostic visit	18	2.42	1.61–3.24	1.39	0.94–1.84
Outpatient screening visit	18	2.95	2.01–3.88	1.62	1.13–2.14
Outpatient visit: taking injections only	12	1.78	1.03–2.53	1.06	0.59–1.54
Inpatient bed day: treatment	7	10.24	1.32–19.15	5.44	0.94–9.94

^*^ US$1 = INR64.456.

CI = confidence interval; ZN = Ziehl-Neelsen; CXR = chest X-ray.

Unit costs of all 50 TB services provided by the study facilities are shown in Supplementary Table S4, while units costs by cost methodology and by input are presented in Supplementary Tables S5A and S5B.

### Variation in TB service unit costs by costing approach and cost driver

In general, top-down (TD) costs were 9% to 109% higher than BU costs, with an average difference of 58%. One exception was culture testing, for which BU cost was 37% higher for liquid media and 41% higher for solid media than TD costs (Supplementary Table S4).

In 30 of 50 TB services, overhead costs were the main driver and on average accounted for 59% (range: 33–98) of the total costs (Supplementary Tables S5A and S5B). Consumables were the major cost driver for 11 of 50 services, and on average this contributed 60% (range: 39–81) of the total costs. Staff cost, which ranged from 42% to 69%, contributed the most for outpatient screening visit, monitoring visit, patient support with voucher, light-emitting diode fluorescence microscopy and hepatitis B surface antigen. Capital cost was the major cost driver for culture in liquid and solid media, the Mantoux tuberculin skin test (TST), inpatient bed days for drug-resistant TB with an average contribution of 48% (range 30–62).

### Unit costs for four TB interventions: vaccination, case-finding, prevention and first-line treatment

Mean costs of four TB interventions are given in Table 3. BCG vaccination required one outpatient visit in each facility and generally one staff nurse was responsible. Average TD cost of BCG vaccination was US$1.16 (standard deviation [SD] 0.53) and BU cost was US$0.76 (SD 0.29). Overall, overhead costs were the highest category of costs in both TD and BU approaches, contributing respectively 34% and 46% to the total costs, followed by capital (BU: 28%; TD: 20%) and staff costs (BU: 27%; TD: 24%).

**Table 3:** TB intervention mean unit costs (2018 US$) ^*^

Intervention	*n*	Top-down		Bottom-up	
	
		Mean	95% CI	Mean	95% CI
BCG vaccination	10	1.16	0.84–1.49	0.76	0.58–0.94
Passive case-finding	18	2.95	2.02–3.88	1.62	1.09–2.14
TB prevention (child <5 years)	5	2.57	1.11–4.03	2.41	1.17–3.65
First-line TB treatment					
Adult EPTB (new and relapse)	11	128.02	92.07–163.97	98.01	73.56–122.47
Adult EPTB (previously treated)	11	252.29	194.52–310.06	182.97	148.22–217.72
Adult PTB (new and relapse)	11	136.15	99.31–172.99	102.77	77.82–127.73
Adult PTB (previously treated)	11	268.73	212.66–324.80	196.03	163.88–228.18

^*^ US$1 = INR 64.456.

CI = confidence interval; BCG = bacille Calmette-Guérin; EPTB = extrapulmonary TB; PTB = pulmonary TB.

Passive case-finding, administered across 18 sampled facilities, involved only one outpatient visit for screening for both PTB and EPTB. Average cost of screening visit was US$1.62 in BU (SD 1.13) and US$2.95 in top-down (SD 2.01). Staff cost was the main cost driver, accounting for 52% of screening visit costs (both TD and BU unit cost), followed by overhead (BU: 34%; TD: 36%) and capital cost (13% in both TD and BU). TB prevention in children under 5 years using isoniazid involved one community visit by the health worker to start the prophylactic treatment and the drug. Average BU cost of the intervention was US$2.41 (SD 1.42) and TD cost was US$2.57 (SD 1.67).

FLT cost in public facilities accounted for inpatient bed days (if required), outpatient visit for collecting medicines, visits for medical follow-up, one sputum smear microscopy at the end of the intensive phase and one at the end of the continuation phase, and in some cases one chest X-ray for PTB patients and first-line treatment drugs. However, the implementation of FLT across sampled facilities varied.^*^ Complete FLT was provided in 11 of 20 sampled public facilities and had a mean BU cost for new PTB cases US$103, and US$98 for EPTB, including US$54 for drugs in each (Table 3). For previously treated cases, BU costs averaged US$196 for PTB (including US$93 for drugs) and US$183 for EPTB (including US$85 for drugs).

## DISCUSSION

This paper estimated unit costs for 50 TB services and four TB interventions using data from 20 randomly selected facilities in two districts from two states in India: Maharashtra and Tamil Nadu. To the best of our knowledge, this is the first study in an Indian context to estimate unit costs for such a wide range of TB services delivered in both public and private health facilities. The unit costs obtained using the TD cost methodology were generally higher than BU estimates; this difference could indicate capacity inefficiencies. This is supported by the evidence that in most TB services (about 59%), overhead expenses were the major cost contributor, pointing to an area for potential future cost reductions to reduce inefficiencies. Understanding efficiency in the application of cost data will be key for the NTEP when using cost data to ensure adequate resource allocation.

Unit costs of TB services estimated in this study were generally lower than those found in other studies conducted in India. The present study reported cost per BCG vaccination at US$0.76 in the BU approach and US$1.16 in the TD approach, much lower than previously published national estimate of US$2.35 (range: 1.41–3.00) per dose for routine immunisation (inflated to 2018 US$).^15^ Probable reasons are inclusion of all antigens, including BCG (not only BCG), outreach vaccinations, above-service costs and incentives linked with full and complete vaccination as per the Indian routine immunisation system in the previous study, all excluded from the present study.

Unit costs of smear microscopy and Xpert testing in the current study using both TD and BU cost methodologies were significantly higher than previously published estimates based on data from five DMCs and two IRL across three states in India.^9^ Mean TD smear microscopy and Xpert costs (converted to 2018 prices) in previous and current study were US$1.63 vs. US$4.08 and US$14.64 vs. US$29.81, respectively. Relatively lower unit costs in the previous study may have been the result of economies of scale from the larger sites included compared to a range of sizes included in the present study. However, both studies identified overhead costs as major cost contributors to smear microscopy costs, and capital and consumables for Xpert testing.

In 2018, FLT costs for PTB adults was generally higher than EPTB FLT costs for both new and previously treated cases. This is because, in almost all facilities, the tests required for EPTB treatment follow-up (e.g., computed tomography scan, ultrasound) were not conducted in those facilities, patients received those tests outside the costed facility (either at higher-level public facilities or at private facilities), and those costs were not captured in this calculation. For every PTB patient, one smear microscopy was performed prior to entering the continuation phase and another after finishing the continuation phase. In some facilities, chest X-rays were also performed during the treatment phase. The smear microscopy tests were not applicable, and X-ray was applicable only in a few EPTB patients; hence, their FLT costs appeared lower.

### Limitations

The following limitations of the study merit comment. First, diagnosis for PTB and EPTB for a child patient was delivered in 25% of sampled facilities, but due to lack of data, modalities and the referral of child patients to specialised facilities for treatment initiation, activities or interventions related to child cases were omitted from the present estimates. Second, six out of 13 public facilities (46%) also offered second-line treatment (SLT) (long regimen) to adult patients during the study period. However, patients were referred for side-effect management, follow-up cultures, additional drug resistance testing and comorbidity management; therefore, cost per patient and episode of SLT could not be calculated comprehensively in this study and partial SLT costs are not reported. However, we were able to cost TB services associated with SLT such as line-probe assays, Xpert testing and testing for adverse event reactions to SLT drugs (liver function test, renal function test, electrocardiogram paper recording, full haemograms). Finally, a significant proportion of staff time data were collected through time sheets (about 54%), a method that is less favoured than observation and interviews in the standard methodology that we applied.^11^ Hence, staff cost estimated using timesheets may have been over or underestimated.

## CONCLUSION

This study responds to the appeal for recent and comprehensive data on TB services and interventions required for current and upcoming scale-up of TB preventive therapy in the South East Asia region,^16^ and other resource needs assessments in India and surrounding regions. Units costs generated from this study can equip planners and economists, understand resource allocation and financial needs using standardised unit costs across key services and interventions delivered to patients affected with TB. Better estimation of investments required for implementing the revised algorithm for prevention, diagnosis and treatment of TB patients should enable India to reach its End TB targets.
